# A facile synthesis of bismuth oxychloride-graphene oxide composite for visible light photocatalysis of aqueous diclofenac sodium

**DOI:** 10.1038/s41598-020-71139-y

**Published:** 2020-08-25

**Authors:** Jamshaid Rashid, Shumaila Karim, Rajeev Kumar, M. A. Barakat, Bilal Akram, Naveed Hussain, Hu Bin Bin, Ming Xu

**Affiliations:** 1 Department of Environmental Sciences, Faculty of Meteorology, Environment and Arid Land Agriculture, King Abdulaziz University, Jeddah, 21589 Saudi Arabia; 2 Department of Environmental Science, Faculty of Biological Sciences, Quaid-I-Azam University, Islamabad, 45320 Pakistan; 3 Key Laboratory of Geospatial Technology for the Middle and Lower Yellow River Regions, College of Environment and Planning, Henan University, Kaifeng, 475004 China; 4 Central Metallurgical R & D Institute, Helwan, 11421 Cairo Egypt; 5 Department of Chemistry, Tsinghua University, Beijing, 100084 People’s Republic of China; 6 State Key Laboratory of New Ceramics and Fine Processing, School of Material Science and Engineering, Tsinghua University, Beijing, People’s Republic of China; 7 Key Laboratory for Special Functional Materials of Ministry of Education, National & Local Joint Engineering Research Centre for High-Efficiency Display and Lighting Technology, School of Materials and Engineering, Collaborative Innovation Centre of Nano Functional Materials and Applications, Henan University, Kaifeng, 475004 People’s Republic of China

**Keywords:** Environmental sciences, Environmental chemistry

## Abstract

In this study, bismuth oxychloride/graphene oxide (BiOCl-GO) composite was fabricated by facile one pot hydrothermal method. The pure BiOCl and BiOCl-GO composite was characterized by X-ray diffraction, Transmission electron microscopy X-ray photoelectron spectroscopy and UV–Vis diffuse reflectance spectroscopy. The synthesized composite was then assessed for photocatalytic degradation of diclofenac sodium (DCF) in visible as well as direct solar light and UV irradiation. Results indicated that the photocatalytic removal efficiency of DCF was significantly affected by dose of catalysts, pH value and source of light. The results reveled that degradation efficiency of BiOCl-GO for DCF reduced from 100 to 34.4% with the increases in DCF initial concentration from 5 mg L^−1^ to 25 mg L^−1^. The solar light degradation of DCF using BiOCl-GO was achieved with apparent rate constant 0.0037 min^−1^. The effect of scavengers study revealed that superoxide ions and holes were mainly responsible for DCF degradation. The regeneration study indicates that BiOCl-GO composite can be successfully recycled up to the five cycles. The study revealed the effectiveness of one pot hydrothermal method for the fabrication of BiOCl-GO composite.

## Introduction

Water pollution is considered a key challenge among major global environmental issues. Emerging organic pollutants include endocrine disrupting chemicals, pesticides, synthetic dyes, and pharmaceutical compounds that enter the environmental compartments through wastewater^1^. Diclofenac (DCF) is widely used as a pain killer primarily for dysmenorrhea, rheumatoid arthritis and inflammation^2^. About 75–150 mg is administered orally as patient’s daily dose, of which nearly 65% passes through the system unaffected or as metabolites^3^. Its tendency to dissolve in water with high polarity and less degradability makes it accumulate in water, hence resulting in water pollution^4^. Therefore, DCF is a worth removing substance from our water environments^5^. Conventional treatment methods such as activated sludge treatment, chlorination, sedimentation, microbial degradation, membrane bioreactors and granular activated carbon may not be suitable treatments for removal of drugs which are non-biodegradable and toxic in nature, thus, a need for advance treatment process like advance oxidation by photocatalysis to eliminate such pollutants completely from wastewater treatment plants^6^.

Bismuth oxychloride is a promising oxyhalide which contains unique electric and optical properties. BiOCl has a band gap of ~ 3.2 eV constructed as a nanostructure^7^ and is an economically viable and environmental friendly catalyst. In BiOCl, the electron–hole separation is facilitated by its indirect transition and opened structure. Furthermore, BiOCl does not contain toxic substances, hence, is extensively explored in photocatalytic degradation of pollutants^8^. Recently, efforts have been undertaken to enhance the separation of charges such as band and facet engineering to induce proactivity of visible light^9–11^. For water treatment, it is necessary to explore the properties of such materials to increase its photocatalytic activity. Graphene is a carbon-based material having sp^2^- hybridized carbon atoms with a honeycomb structure^12^. Graphene’s composites have been used for water splitting through photocatalysis to generate hydrogen as well as for photocatalytic degradation of pollutants due to its high specific surface area and greater mobility of charge particles^13–16^. Several studies proved that BiOCl is a good photocatalyst but due to fast recombination of photogenerated electron–hole (e^−^/h^+^) pairs, its photocatalytic efficiency is compromised^17–22^. Therefore, coupling of BiOCl with GO could be a facile method to separate the photogenerated e^−^/h^+^ to enhance the visible light photocatalysis properties. Several binary, ternary and quaternary composites based on GO and BiOCl have been reported in the literature which showed the enhanced photocatalytic activity. Lin et al.^12^ synthesized the GO/BiOCl thin film for the decomposition of rhodamine B and almost 99% degradation was observed within 90 min. A reduction in the bandgap energy to 2.9 eV was observed due to coupling of GO with BiOCl in comparing to pure BiOCl (3.2 eV). Zhang et al.^19^, synthesized the 3D BiOCl/RGO aerogel photocatalyst for the adsorption and photocatalytic degradation of oxytetracycline. The BiOCl/RGO aerogel showed 1.43 times higher removal of oxytetracycline than pristine BiOCl under the visible light irradiations. A series of quaternary BiO_x_Cl_y_/BiO_m_Br_n_/BiO_p_I_q_/GO composites have been synthesized by hydrothermal method and used as a visible light active catalyst (Eg − 2.18 eV) for the photocatalytic degradation of 2-hydroxybenzoic acid and O_2_^·^ˉ, h^+^, and ^·^OH were responsible of mineralization of the 2-hydroxybenzoic acid^20^. These studies revealed that the coupling to the semiconductor catalyst with GO enhance the interaction of the composite with pollutant and facilitates efficient decomposition of the organic contaminants in wastewaters.

Herein, BiOCl coupled GO composite was synthesized by facile one pot method for the photocatalytic degradation pharmaceutical DCF pollutant in aquatic system under of UV, synthetic visible and solar light irradiation. The role of solution pH, initial DCF concentration and BiOCl-GO mass was evaluated to identify the optimum photocatalysis conditions. The kinetics and photocatalysis mechanism of DCF degradation onto BiOCl-GO were also investigated.

## Materials and methods

### Chemicals and instrumentation

Chemicals used in this research included diclofenac sodium (DCF), sulfuric acid (H_2_SO_4_), hydrochloric acid (HCl), Sodium bismuthate (NaBiO_3_), hydrogen peroxide (H_2_O_2_), graphite, potassium permanganate (KMnO_4_) and sodium hydroxide (NaOH) were purchased from Sigma Aldrich (USA). Deionized water was used to make stock and working solutions.

### Synthesis of GO, BiOCl and BiOCl-GO

A facile one pot hydrothermal method was used for the synthesis of BiOCl-GO composite. Initially, 60 mg GO was dissolved in 200 mL of 0.6 M HCl solution and stirred for 2 h. Thereafter, 4.0 g NaBiO_3_ was added to the GO solution and left for 16 h for continuous stirring at 25 °C. The obtained materials were transferred into hydrothermal reactor and heated at 140 °C for 16 h. The obtained material was washed with deionized water, acetone and ethanol to remove the excess amount of HCl and dried at 105 °C for 8.0 h. A similar method was used for the synthesis of BiOCl in the absence of GO. Hummer’s method was used for the preparation of GO^23^.

### Characterization

XRD analysis was performed to find out the crystalline nature of catalysts BiOCl and BiOCl-GO on Bruker axis D8 model. Transmission electron microscopic (TEM) analysis was conducted on HITACHI-H-770 with acceleration voltages of 100 kV. X-ray photoelectron spectroscopy (XPS) analysis was accomplished on standard Omicron system equipped with monochromatic Al Kα 1,486.7 eV X-ray source operated at 15 keV. Absorption spectrometry was performed using Shimadzu UV-2550 UV–Vis spectrophotometer.

### Photocatalytic experiments

The photocatalytic experiments were accomplished using BiOCl and BiOCl-GO composite in visible light. For that purpose, 100 mL of different concentrations of DCF (5, 15 and 25 mg L^−1^) were taken in 250 mL Pyrex breaker and added 1.0 g L^−1^ of the catalyst. Before exposure to light, adsorption–desorption for 30 min achieved by stirring DCF and catalyst solutions. The DCF molecules were photocatalytically degraded using different light sources including UV mercury lamps (12 lamps × 8 W each), white cool visible lamps (spectral irradiance 17.45 mW cm^−2^; λ = 390–700 nm) and visible region of solar light (intensity 22,000 lx ≈ 17.38 mW cm^−2^). The suspension was then exposed to respective light sources for 180 min under continuous stirring throughout the experiment, 5 mL aliquots of DCF solution were taken out after the intervals of 30 min and analysed using UV–Vis spectrophotometer at λ_max_ 276 nm. Percentage degradation of DCF was calculated by applying following equation:

1 $$DE \%=\left(\frac{{C}_{o}-{C}_{t}}{{C}_{0}}\right)\times 100$$

where C_o_ refers to initial concentration of DCF while C_t_ refers to concentration of DCF at time t.

## Results and discussion

### Characterization

**Figure 1:**
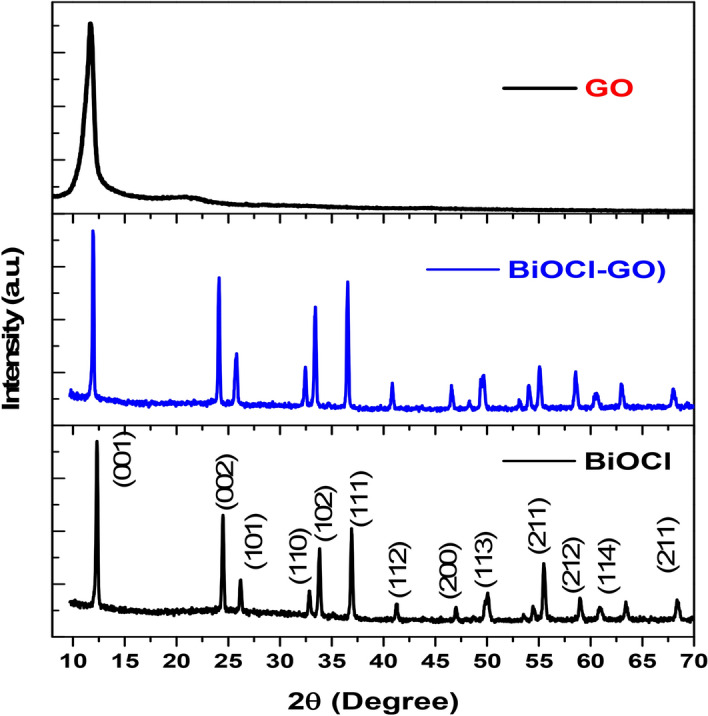
XRD pattern of GO, BiOCl and BiOCl–GO*.*

The XRD patterns of BiOCl and BiOCl-GO are shown in Fig. 1. Results suggested that diffraction peaks of BiOCl were coinciding with standard (JCPDS # 00-006-0249) reference material^21^. The result revealed a highly pure tetragonal structure for BiOCl. Strong characteristic peak appeared at 11.75° which was indexed as (001), shown in layers of [Cl–Bi–O–Bi–O–Cl] stacking structure present along c-axis^20^. The XRD pattern of BiOCl–GO composite is almost same as BiOCl with slight change in the peak position and intensity. Generally, GO characteristic peak for (002) appeared at 11.7° which is overlapping with the BiOCl peak (001) at 11.75 in BiOCl-GO composite ^24^.

**Figure 2:**
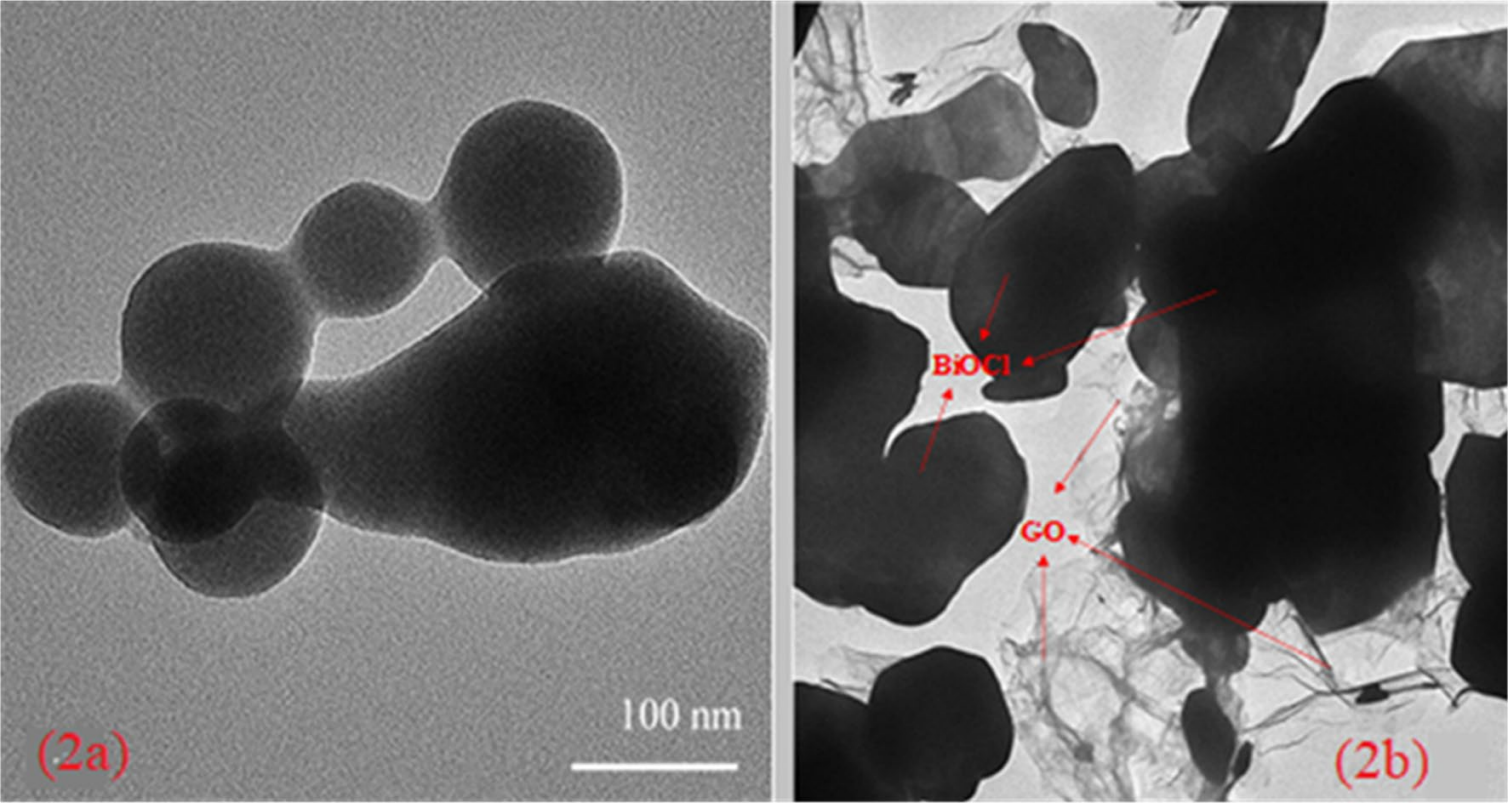
TEM images of (**a**) BiOCl and (**b**) BiOCl–GO composite.

The morphology of BiOCl and BiOCl–GO were identified with the help of Transmission electron microscopy. It was clearly indicated in Fig. 2a, that BiOCl particles are sheet like structures. Figure 2b illustrates the well distibuted GO sheets over BiOCl. Sun^21^ also synthesized distinct plate like BiOCl from solvothermal process having width ranged from 150–300 nm. However, in BiOCl-GO composite, GO sheets are found to be wrinkled on the outer edges embedded by BiOCl Fig. 2b. TEM analysis revealed the sucessful synthsis of BiOCl-GO composite.

**Figure 3:**
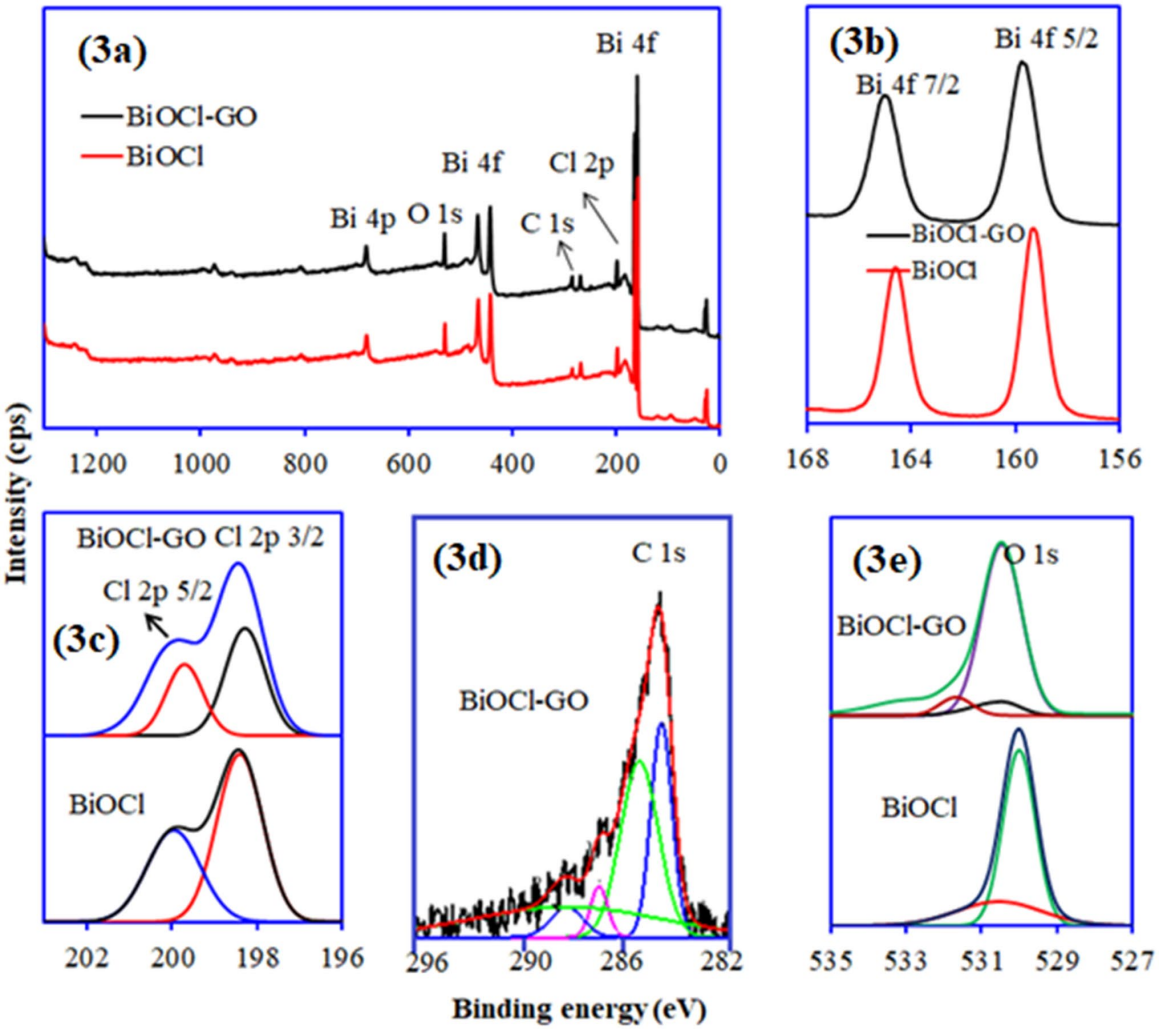
XPS analysis of BiOCl and BiOCl/GO composite (**a**) wide scan survey and deconvoluted spectra for (**b**) Bi 4*f *(c) Cl 2*p*, (**d**) C 1*s* and (**e**) O 1*s.*

XPS analysis of BiOCl and BiOCl–GO composite is showed in Fig. 3a. The survey scan spectra confirmed the presence of Bi, O, Cl and C. The peak at 159.3 eV was ascribed for Bi 4*f*, 198.05 eV for Cl 2*p*, 530.01 eV for O 1*s* and 284.78 eV attributed for (adsorbed carbon) C 1*s*. The presence of C as an impurity came from the atmospheric carbon dioxide^20^. The XPS analysis results revealed that atomic carbon content in BiOCl and BiOCl-GO composite was 28.68 and 38.3%, respectively. The associated resolved peaks for Bi 4*f*, Cl 2p, C 1*s* and O 1*s* are provided in Fig. 3b–e respectively. The binding energy values, and atomic percentages of the identified elements are also listed down in Table 1. The peak located at 159.71 eV corresponds to Bi 4*f* in the BiOCl–GO composite^25^. In comparison with the pure BiOCl, the Bi 4*f* peak of BiOCl–GO is shifted towards a slightly higher binding energy due to the interaction among BiOCl and GO^25,26^. In BiOCl–GO composite, the peak for Cl 2*p* was identified at 198.5 eV, belongs to the characteristic peak of Cl^−^in BiOCl. Similarly, O1*s* peak of BiOCl–GO composite was identified at 530.47 eV, corresponding to O^2−^ of Bi–O bond in BiOCl. Furthermore, the peak recognized at 284.8 eV was ascribed for C 1*s* in BiOCl–GO which indicated C–C bond with *sp*^2^ orbitals^26^. A difference in the binding energy about 5.3 eV and 5.31 eV was observed between Bi 4*f*_5/2_ and Bi 4*f*_7/2_ in both BiOCl and BiOCl-GO composite, respectively, which is revealing the presence of Bi in the chemical state of Bi^3+^ in both materials^21^.

**Table 1:** Elemental composition of BiOCl and BiOCl-GO (XPS analysis).

Element	BiOCl		BiOCl-GO	
	BE (eV)	Atomic (%)	BE (eV)	Atomic (%)
Bi 4*f*	159.3	20.06	159.71	16.8
Cl 2*p*	198.05	25.24	198.5	21.2
C 1*s*	284.78	28.68	284.8	38.3
O 1*s*	530.01	26.02	530.47	23.7

**Figure 4:**
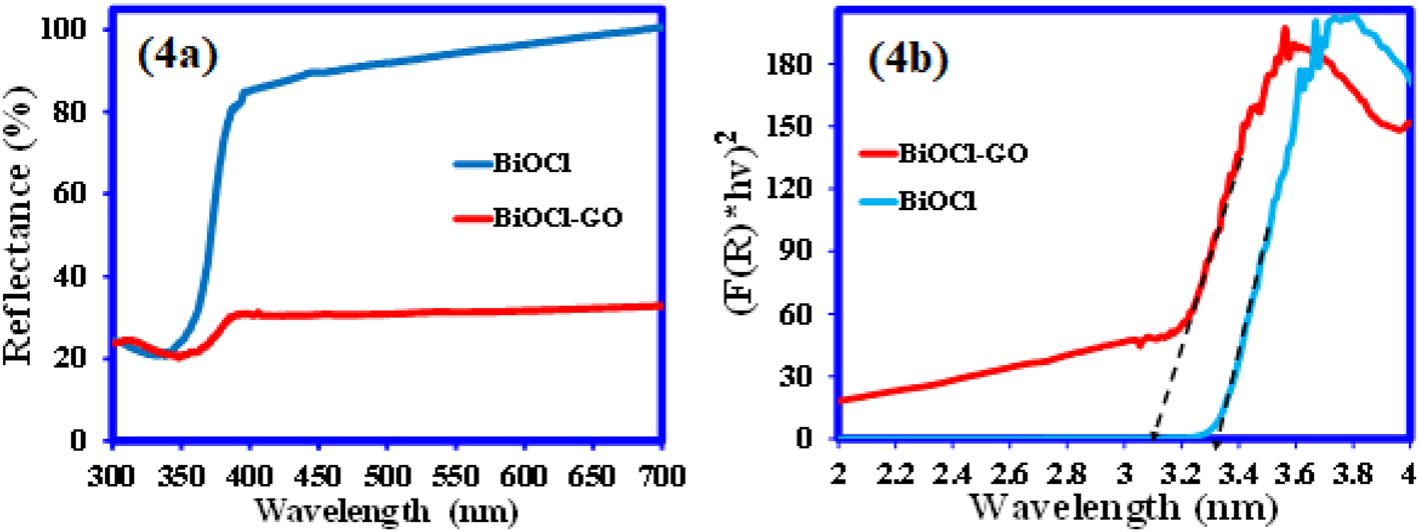
(**a**) UV–Vis diffuse reflectance spectra and (**b**) Kubelka–Munk plot of BiOCl and BiOCl–GO composite.

The light absorption propreties of the BiOCl and BiOCl–GO composite were detrmined using UV–Vis diffuse reflectance spectroscopy. The UV–Vis DRS (Fig. 4a) shows that pure BiOCl has very strong absorption in the UV region with a sharp edge around 372 nm. While BiOCl–GO composite showed the greater improvement in the absorption of the light in the visible region. As shown in Fig. 4a, a red shift in the absorption edge of BiOCl-GO composite is detected in comparison to pure BiOCl^11^. These results reveleing that couplong the GO with BiOCl enhaced the optical absorption properties of pure BiOCl and a bnd gap shift from 3.33 to 3.08 eV was observed (Fig. 4b).

### Photocatalysis

**Figure 5:**
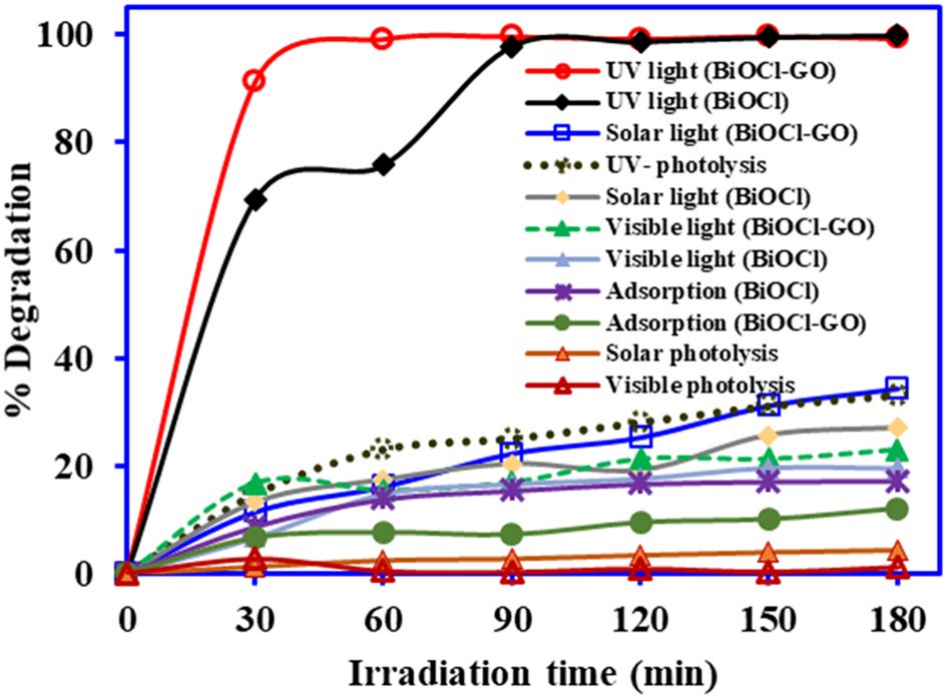
Comparative adsorption, photolysis and photocatalytic degradation of DCF on BiOCl and BiOCl-GO (catalyst mass—1 g L^−1^, DCF conc.—25 mg L^−1^, vol.—100 mL)*.*

**Figure 6:**
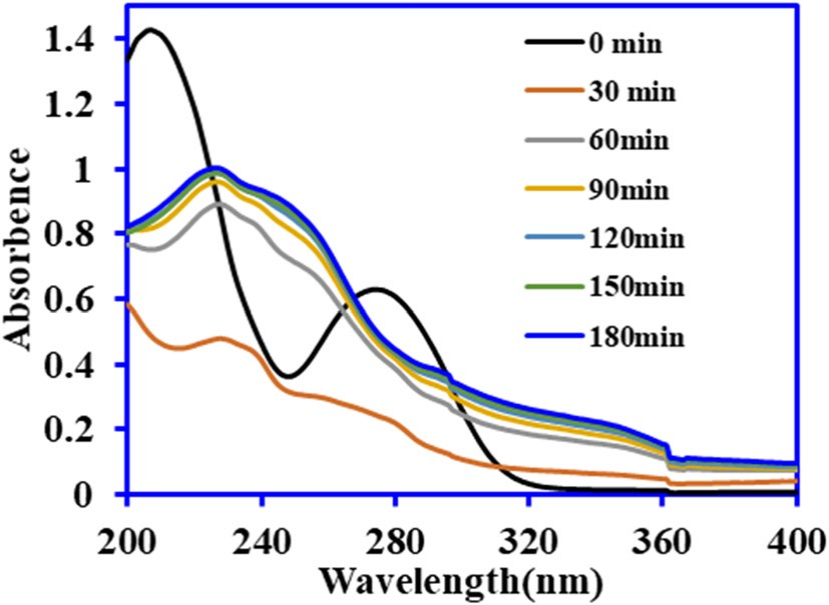
Photolytic degradation of DCF under UV irradiation. (DCF conc.—25 mg L^−1^, vol.—100 mL, time—180 min)*.*

The adsorption experiment was conducted in dark for 3 h using BiOCl and BiOCl-GO catalysts. Figure 5 shows that about 17.2% and 11.9% of the pollutant were removed within 60 min by BiOCl and BiOCl–GO, respectively. Thereafter, equilibrium was established, and no further adsorption was observed. The results were similar to the study conducted by Mendez et al.^27^, where only 14% of DCF was removed by TiO_2_. Another study was in a close agreement with the finding where 5% of DCF was adsorbed onto the catalyst in first 30 min and the adsorption remained constant for the next 240 min^28^. The photolysis of DCF under UV, and solar irradiation was evaluated without adding the catalyst for three hours and the results depicted in Fig. 5 suggested that under visible light the photolysis of DCF was negligible while UV light degrade almost 33% DCF. Similar results were observed by Zhang^29^ who reported negligible degradation of DCF under visible light. In UV photolysis, the concentration of DCF decreases gradually and the characteristic peak at 276 nm was shifted to 240 nm after 30 min exposure to UV light. With the increase in exposure time up to 180 min, the absorbance was also increased with various peaks within wavelength ranging from 230–250 nm as shown in Fig. 6. The shifting of peak from 276 nm revealed the formation of intermediate products. Di Credico^30^ observed the peaks for DCF at wavelength 210, 240, 289, and 324 nm which were due to production of carbazole compounds that were formed by loss of Cl atom from DCF parent molecule. Several studies have reported that intermediate molecule such as hydroxylated and mono-halogenated carbazole compounds are generated in photolysis process for DCF pollutant. Such intermediates are persistent in water. The appearance of light reddish-brown color of DCF solution with the light exposure for 180 min reveals the formation of intermediates^3,31,32^.

Figure 5 shows the BiOCl/DCF photocatalysis in UV light with complete degradation within 90 min while BiOCl–GO/DCF photocatalytic degradation was completed within 60 min. These results revealed that incorporation of GO with BiOCl played a positive role in the photocatalysis. However, the DCF degradation using BiOCl–GO under solar light and visible light irradiation was 34.5% and 22.9%, respectively. The large difference in the photocatalytic efficiency of the BiOCl-GO composite under UV, solar and synthetic visible light is mainly due strong photolytic interaction of DCF with UV light. The UV light (λ_max_ – 254 nm) energy is much higher than visible light (λ_max_ > 400 nm). At greater light intensity, the production of electron hole pair generation is higher, and a large amount of reactive species are generated which are involved in degradation^33–35^. Moreover, UV light photolysis (without catalyst) degrades almost 34% DCF which is also responsible for higher DCF degradation under UV photocatalysis process. Therefore, BiOCl and BiOCl-GO showed higher DCF degradation in UV irradiation in compassion to solar and synthetic visible light. Although, cost and safety hazards of UV light in the catalytic process mandate that natural solar light photocatalysis is a viable option. Therefore, experimental conditions must be optimized to find the optimum photocatalytic degradation of DCF using BiOCl-GO composite in the solar light.

**Figure 7:**
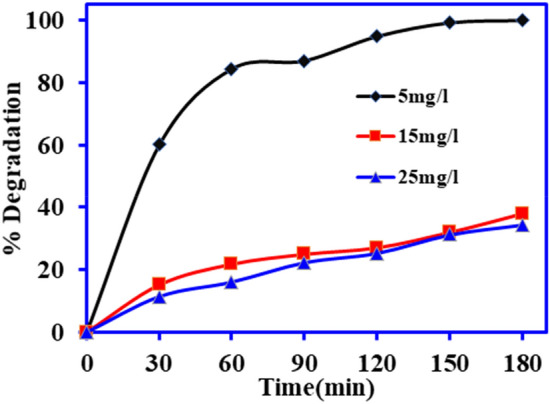
Role of initial DCF concentration onto photocatalysis using BiOCl-GO composite under solar light irradiation. (pH 6, vol.—100 mL, time 0–180 min, catalyst dose = 1.0 g L^−1^)*.*

The effect of DCF concentration onto the photocatalysis using BiOCl-GO composite was studied at 5, 15 and 25 mg L^−1^ and the results are depicted in the Fig. 7. The results showed that the degradation rate is inversely proportional to initial concentrations. About 100%, 38.1% and 34.3% degradation were observed at 5, 15 and 25 mg L^−1^ concentrations, respectively. With the increase in DCF initial concentrations, the removal rate decreased due to lower photon absorption by the photocatalyst because of the magnified number of DCF molecules that hampers the active radical production, hence decreasing the degradation efficiency^1^. Additionally, when the substrate concentration is higher, the surface of the BiOCl-GO composite may be accumulated by the DCF molecules that could result in the reduced surface area for degradation of the pollutant^36^.

**Figure 8:**
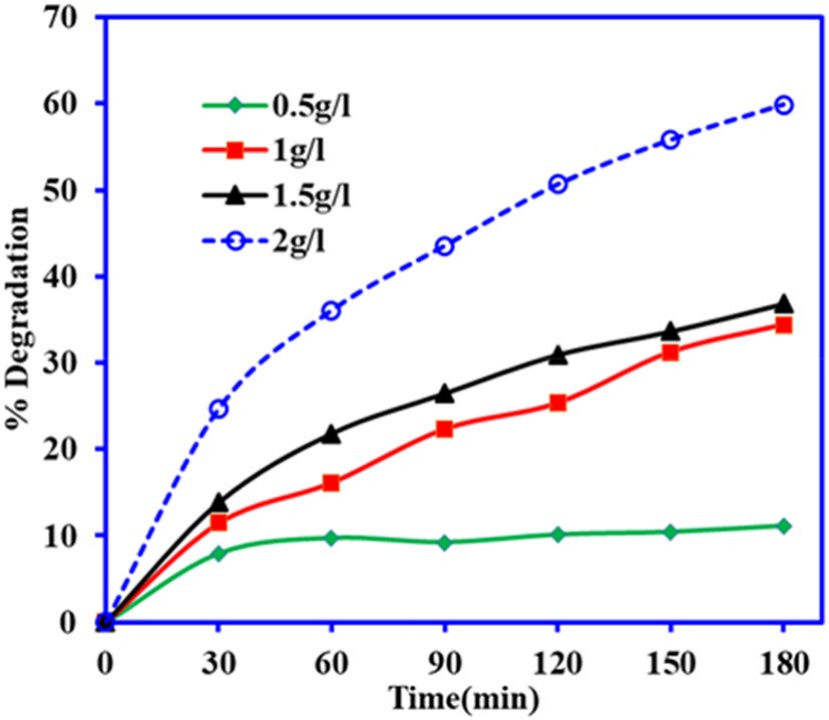
Effect of catalyst mass on the photocatalytic activity of BiOCl–GO composite on DCF (conc. 25 mg L^−1^, pH 6, vol.—100 mL).

The mass of catalyst is one vital parameter which regulates the efficiency of the catalysis process. The degradation of DCF was increased with the increase in mass of BiOCl–GO photocatalyst as shown in the Fig. 8 and 11.1%, 34.3%, 36.7% and 50.8% of DCF photodegradation was found at 0.5, 1, 1.5 and 2 g L^−1^ BiOCl–GO mass, respectively. The degradation behavior due to increased catalyst load may be ascribed to the presence of greater number of active sites for pollutant along with the higher absorbance of light by BiOCl–GO catalyst resulted in the generation of higher number of reactive species (super oxide and hydroxyl species) that are responsible for photocatalytic degradation^37^.

**Figure 9:**
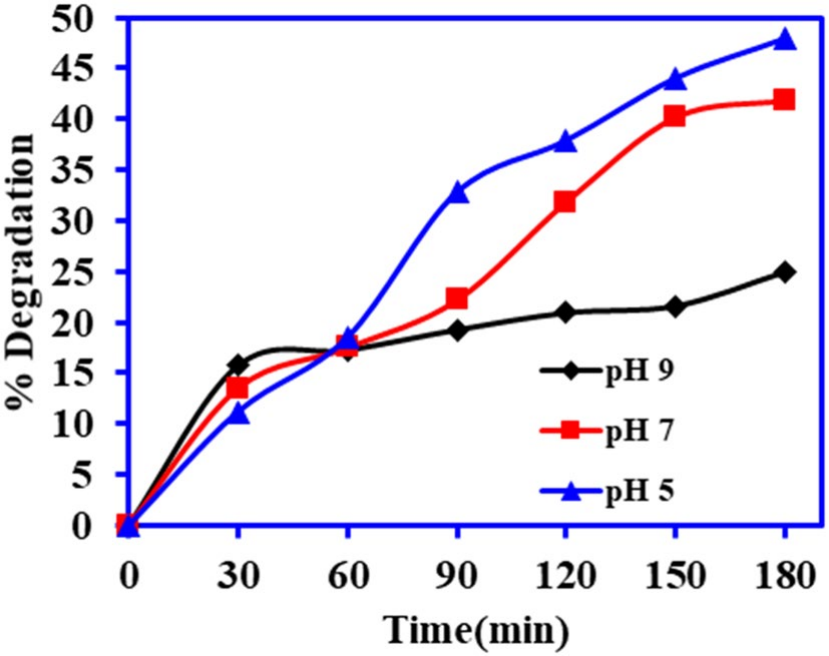
Role of solution pH on the photocatalytic activity BiOCl-GO catalyst (catalyst mass—1.0 g L^−1^, vol.—100 mL, conc. 25 mg L^−1^, light source—sunlight).

In the photocatalytic processes, solution pH plays a pivotal role due to the change in the surface charge and functionality of the catalyst and pollutants. Herein, the degradation of the DCF on to BiOCl-GO composite was investigated at the pH 5, 7 and 9 pH because DCF is poorly soluble in water at the lower pH (below pH 3)^38,39^. The results depicted in Fig. 9, revealing that acidic medium is the most suitable for the photocatalytic decomposition of the DCF and maximum degradation was observed at pH 5. The higher degradation of DCF at slightly acidic pH is due to the good interaction between the protonated BiOCl-GO composite and the anionic DCF (pKa—4.2)^39^. Conversely, the presence of anionic DCF in alkaline medium and the negatively charged catalyst resists the interaction of DCF, results in low photocatalytic degradation as shown in the Fig. 9.

The heterogeneous photocatalysis degradation of DCF was found to be more relevant to kinetic model pseudo-first order as shown in Eq. 2^40^.

2 $$ln\left(\frac{C}{{C}_{^\circ }}\right) =-kt$$

where, C represented as concentration of DCF at time t, C_o_ denotes as initial concentration of DCF and *k* (min^-1^) is apparent rate constant. Kinetic plots for each parameter (initial concentration, dose, and pH) were obtained by ln(C_0_/C_t_) vs time (Fig. S1). Apparent rate constant values obtained from plots and R^2^ values are represented in Table 2. The values of *k* for different mass of BiOCl-GO composite showing inverse relation among rate constant and initial DCF concentration. The results revealed that with the increase in catalyst dose of BiOCl-GO from 0.5 to 2 g L^−1^, the apparent rate constant increases from 0.0003 to 0.0042 min^-1^, respectively. For varied pH conditions the highest rate constant was observed at pH 5 with the value of 0.0037 min^−1^. These results suggested that the rate of DCF degradation was higher in acidic condition.

**Table 2:** The kinetics data of DCF degradation by BiOCl-GO for initial DCF concentration, catalyst mass and solution pH.

Parameters	Sample name	Pseudo first order (BiOCl–GO)	
		*k* (min^−1^)	R^2^
Initial concentration	5 mg L^−1^	0.0386	0.9074
	15 mg L^−1^	0.0018	0.9601
	25 mg L^−1^	0.002	0.9943
Dose of catalyst	0.5 g L^−1^	0.0003	0.899
	1.0 g L^−1^	0.002	0.9943
	1.5 g L^−1^	0.002	0.9778
	2.0 g L^−1^	0.0042	0.993
pH of solution	pH 5	0.0037	0.9784
	pH 7	0.0029	0.9647
	pH 9	0.0007	0.9701

### Photocatalysis mechanism

**Figure 10:**
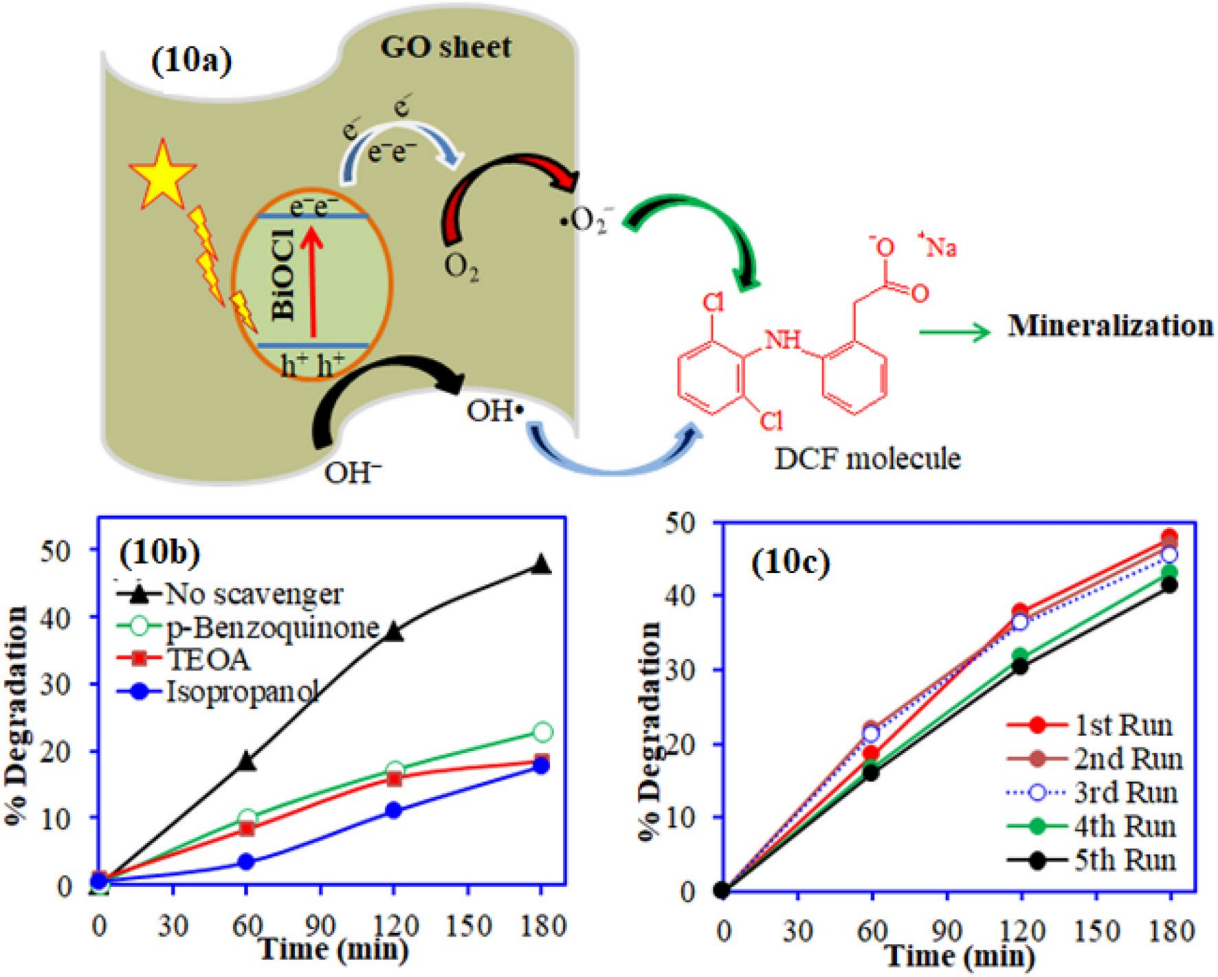
(**a**) Schematic representation for DCF photocatalytic degradation mechanism onto BiOCl–GO composite; (**b**) Effect of different scavengers on DCF degradation and (**c**) Plot for regeneration of spent BiOCl–GO composite.

The photocatalytic degradation mechanism of DCF on to BiOCl-GO catalyst is shown in Fig. 10. The direct band gap energy of the BiOCl was found to be around 3.33 eV as reported in other studies^17^. Due to large band gap energy, BiOCl is not visible light active catalyst. After incorporation of BiOCl with GO, the composite showed band gap shifts to 3.08 eV exhibiting good catalytic properties. Under the solar light illumination, the photo-induced electrons from the conduction band of the BiOCl moved to its valance band^41^. Thereafter, these electrons are trapped by the graphene oxide which restricts the electron–hole recombination in the catalyst. The photogenerated electrons and holes are responsible for the production of active radical spaces as shown in Fig. 10a, which are in turn responsible for the decomposition and mineralization of the DCF in the solution.

Moreover, to identify the role of the reactive species i.e., electrons, holes, superoxide radicals and hydroxyl ions on the DCF degradation, the photocatalysis experiments were conducted in the presence of scavengers. The p-benzoquinone (PBQ), isopropanol (IP) and triethanolamine (TEOA) were employed for superoxide, hydroxyl radicals and holes scavengers, respectively. The photocatalyst without any scavenger showed around 49% degradation of DCF in sunlight. Adequate amount of TEOA was added to different replicates of DCF solution containing the photocatalyst which exhibited decline in photocatalytic activity up to 18.5%. Similarly, the addition of IP and PBQ also diminished the photocatalytic activity of BiOCl–GO in the order no-scavenger > PBQ > TEOA > IP as shown in Fig. 10b. The significant decline in the photocatalytic activity with addition of BQ, TEOA and IP suggest that the photocatalysis of DCF over BiOCl–GO is affected by holes, super oxides and hydroxyl radicals with major contribution from superoxide ions and holes ^42,43^.

### Reusability of BiOCl-GO composite

The stability and reusability of the spent BiOCl–GO composite was studied up to the five cycles. Prior to the regeneration study, the spent BiOCl–GO composite was washed with de-ionized water and dried at 105 °C. The results for the regeneration studies are depicted in Fig. 10c. The obtained results revealed that a slight reduction in the photocatalytic efficiency of the BiOCl–GO composite for DCF after the fifth cycle. These results clearly demonstrated that BiOCl–GO composite is stable and can be reused for the multiple times for DCF degradation.

### Comparative photocatalysis

To estimate the efficiency of the BiOCl–GO composite, its photocatalytic activity was compared with literature studies. The highest solar light photocatalytic activity for 25 mg L^−1^ Diclofenac was achieved with BiOCl–GO composite compared with previously studies, as shown in Table 3. These results reveal that BiOCl can effectively remove Diclofenac from water. The higher percentage activities of other catalysts may be attributed to either exposure to UV light where photolysis plays a stronger role than photocatalysis^27^ or variation in operational conditions such as lower initial pollutant concentration or use of noble metal dopants^44,45^.

**Table 3:** Comparison with literature studies for photocatalytic degradation of diclofenac.

Photocatalyst	Pollutant	Initial conc.(mg/L)	Removal (%)	conditions	Ref.
BiOCl–GO	Diclofenac	25	47.88	Visible spectrum solar light (≈ 17.38 mW cm^−2^), pH 5, 180 min	This study
TiO_2_	Diclofenac	200	75	Xe lamp (1,000 W, 290–400 nm); time 120 min	^27^
Ni–TiO_2_	Diclofenac	15	47	Solar UV lamp (30 W, 450 nm); time 120 min	^46^
WO_3_	Diclofenac	0.5	100	Xe lamp (1,500 W, 300 nm); time 2 h	^47^
TiO_2_–P25	Diclofenac	2	100	Blacklight philips TLK 05 (40 W, 290–400 nm); 60 min	^44^
Ce-ZnO	Diclofenac	2	100	Blacklight philips TLK 05 (40 W, 290–400 nm); 30 min	
TiO_2_–SG	Diclofenac	2	100	Blacklight philips TLK 05 (40 W, 290–400 nm); 30 min	
C_3_N_4_	Diclofenac	10	19.3	Xe lamp (300 W, ≥ 400 nm), 60 min	^48^
CNC2-C_3_N_4_	Diclofenac	10	100	Xe lamp (300 W, ≥ 400 nm) 60 min	
TiO_2_–FeZ/H_2_O_2_	Diclofenac	30	42.5	Xe lamp (450 W), 180 min	^49^
BiOCl–Au–CdS	Sulfadiazine	20	100	Xe lamp (300 W), 240 min	^45^
BiOCl	Sulfadiazine	20	86.2	Xe lamp (300 W), 240 min	

## Conclusion

It is concluded from this study that BiOCl and BiOCl–GO were successfully synthesized by a facile one pot hydrothermal method. The photocatalytic activity of BiOCl–GO was systematically examined for the degradation of micro pollutant diclofenac sodium. In comparison to BiOCl, the BiOCl–GO composite showed complete degradation of DCF within 60 min in UV light for 25 mg L^−1^ DCF. Solar and synthetic visible light mediated experiments also favored superior activity of BiOCl–GO towards DCF with higher pseudo first order kinetic constant value 0.0386 min^−1^. The cost and safety hazards of UV light in the catalytic process mandate that natural solar light photocatalysis a viable option. This study showed a great potential to degrade pharmaceutical pollutants under solar irradiation for enhanced removal of micropollutants from the aqueous environments.

## Supplementary information

Supplementary Information.
