# Supplementary material for: A facile synthesis of bismuth oxychloride-graphene oxide composite for visible light photocatalysis of aqueous diclofenac sodium
Source: Sci Rep. 2020 Aug 25;10:14191. doi: 10.1038/s41598-020-71139-y (PMC7447784; doi:10.1038/s41598-020-71139-y)
Supplement: Supplementary file 1 — Supplementary Information. [file 41598_2020_71139_MOESM1_ESM.docx]

A facile synthesis of bismuth oxychloride-graphene oxide composite for visible light photocatalysis of aqueous diclofenac sodium

**Supplementary Data:**

**Figure S1**: Pseudo First order kinetic Plots for DCF Photocatalysis by BiOCl-GO
